# Transcriptional changes across tissue and time provide molecular insights into a therapeutic window of opportunity following traumatic stress exposure

**DOI:** 10.1101/2024.11.01.621484

**Published:** 2024-11-03

**Authors:** Lauren A McKibben, Meghna R Iyer, Ying Zhao, Roxana Florea, Sophia Kuhl-Chimera, Ishani H Deliwala, Yue Pan, Erica M Branham, Sandrine M Geranton, Samuel A McLean, Sarah D Linnstaedt

## Abstract

Unfortunately, survivors of traumatic stress exposure (TSE) frequently develop adverse posttraumatic neuropsychiatric sequelae (APNS) such as chronic pain and stress/depressive symptoms. Increasing evidence indicates that there is a 'window of opportunity' following TSE in which therapeutic interventions are most effective against APNS, yet mechanisms accounting for this observation are poorly understood. Here, we aimed to better understand such mechanisms by generating snapshots of the transcriptional landscape in the early aftermath of TSE across tissues and time. Adult rats were exposed to a TSE model, single prolonged stress (SPS). Then, eight tissues (hypothalamus, left and right hippocampus, amygdala, dorsal root ganglia, spinal cord, heart, and muscle) were isolated from these animals at 2, 24, and 72 hours after SPS and in unexposed controls (n=6 per group). mRNA expression from deep sequencing was used to identify differentially expressed genes (DEGs) and biological pathways enriched over time. In all tissues except the amygdala, the highest number of DEGs was observed 2-hours post-SPS, but DEGs were detected at all timepoints and in all tissues. Some transcripts were differentially expressed in a consistent manner across multiple tissues at a time point (e.g. Fkbp5, 2 hours post-SPS), while others had tissue- or region-specific expression patterns. Stress system pathways were most represented at 2-hours post-SPS, then stress/circadian/inflammatory pathways at 24-hours, and inflammatory pathways at 72-hours. Together these findings provide insights into post-TSE transcriptional landscape dynamics and suggest specific intervention windows of opportunity. Future validation is needed across sex, age, stressor, and cell type.

## Full Text Availability

The license terms selected by the author(s) for this preprint version do not permit archiving in PMC. The full text is available from the preprint server.

